# Long-Term Follow-Up Results of a Patient Undergoing Acute Retinal Necrosis: A Case Report and Literature Review

**DOI:** 10.1155/2021/9997155

**Published:** 2021-07-10

**Authors:** Büşra Köse, Hidayet Erdöl, Dilek Uzlu

**Affiliations:** ^1^The State Hospital of Bayburt, Bayburt, Turkey; ^2^Karadeniz Technical University, Medical Faculty, Department of Ophthalmology, Trabzon, Turkey

## Abstract

**Purpose:**

To describe the follow-up and treatment approach of a patient with acute retinal necrosis. *Case Report*. A 44-year-old male patient, who had complaints of pain in the right eye and blurred vision, was referred to our clinic. Best-corrected visual acuity (BCVA) was 0.4. There was 2+ anterior chamber reaction and diffuse smooth-rounded keratic precipitates. Fundus examination revealed optic nerve and vascular involvement. Fundus fluorescein angiography revealed extensive ischemia in the periphery. Oral antiviral therapy was preferred. In addition, systemic steroid and laser photocoagulation were applied. Nonetheless, retinal detachment developed 2 months later. Detachment, silicon removal, and cataract surgery were performed sequentially. It was observed that the patient was followed for 4.5 years and did not have contralateral eye involvement. Best-corrected visual acuity remained stable at 0.3.

**Conclusion:**

Early diagnosis, appropriate treatment, accurate complication management, and frequent follow-up may provide useful vision in patients with acute retinal necrosis.

## 1. Introduction

Acute retinal necrosis is often unilateral, characterized by well-demarcated areas of necrosis in the retina deriving from Varicella zoster virus (VZV) or Herpes simplex virus (HSV) [1–4]. This entity is infectious uveitis in which vitreous and anterior chamber inflammation is added to the manifestation with retinitis exhibiting rapid and circumferential progression leading to occlusive retinal vasculopathy also involving the arteries [5, 6]. More rarely, cytomegalovirus and Epstein-Barr virus have also been shown to be capable of leading to the manifestation [4]. Visual outcomes are generally poor, with visual acuity decreasing to below 20/200 six months after onset in 48% of patients [7].

Treatment currently consists of intravenous acyclovir at a dosage of 10 mg/kg three times daily for 7-10 days, followed by long-term oral acyclovir therapy [3, 4, 8, 9]. Although antiviral therapy reduces the rate of involvement in the contralateral eye, the risk of severe loss of vision and retinal detachment in the affected eye is still high [3, 4, 7, 9–12]. Response to treatment varies from patient to patient, and the effect cannot be predicted.

Due to the high rates of acyclovir-related side effects, new antiviral drugs such as the prodrug valacyclovir, with higher oral bioavailability and a lower incidence of side effects, have been employed in clinical practice in recent years [11, 13, 14].

In this case report, we describe the clinical course of acute retinal necrosis in a patient under long-term follow-up.

## 2. Case

A 44-year-old man presented to our clinic due to reduced vision in May 2016. No characteristic associated with the manifestation was present in the patient's own or family history, and he had no history of chronic drug use or comorbid conditions such as severe influenza infection.

**Figure 1:**
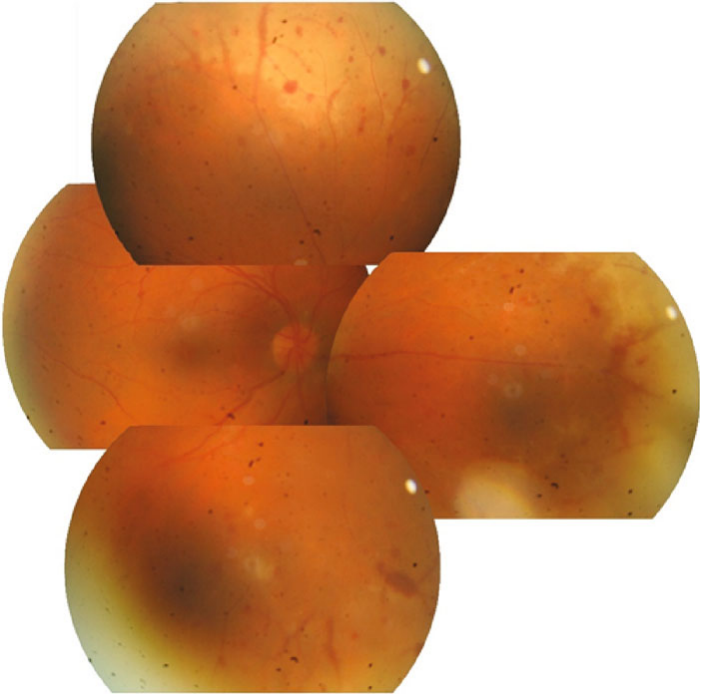
Montage of retinal photographs (Canon 60UVi, Tokyo, Japan) showing vitritis, areas of retinitis, retinal vasculitis, retinal hemorrhage, and hyperemia and edema in the head of the optic nerve at initial presentation examination.

**Figure 2:**
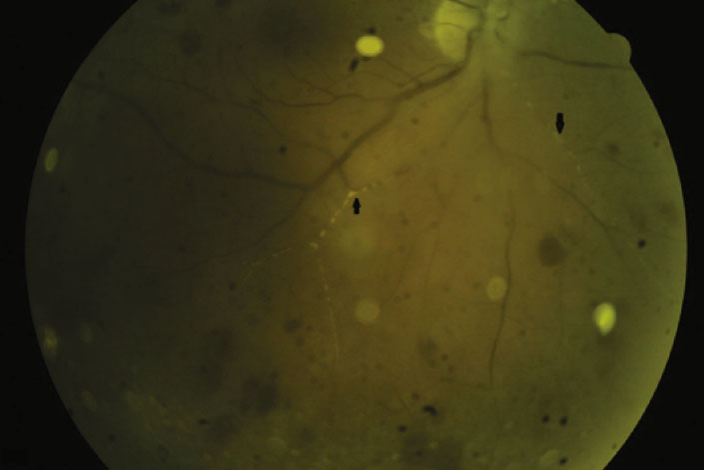
Red-free fundus photograph (Canon 60UVi, Tokyo, Japan) showing occlusive vasculopathy (black arrows).

The patient was referred to us from another clinic with a preliminary diagnosis of uveitis with pain and blurred vision in the right eye for the previous week. Examination using a Snellen chart revealed visual acuity of 0.4 in the right eye. 2+ anterior chamber reaction was present in the anterior segment with diffuse smooth-rounded keratic precipitates. Fundus examination revealed edema and hyperemia in the head of the optic disk, 360-degree retinal hemorrhages in the peripheral retina, sheathing and occlusion of the vessels, and 2+ vitreous cells (Figures 1 and 2). Fundus fluorescein angiography (FFA) (Canon 60UVi, Tokyo, Japan) revealed ischemic areas in front of the equator in the peripheral retina and leakage associated with permeability impairment in vessels. No significant pathology was determined in the macular region on optical coherence tomography (OCT) (Optovue RTVue, RT 100, software version 6.3, Optovue, CA, USA). Acute retinal necrosis was diagnosed, and the patient was started on oral valacyclovir 1000 mg at 8 h intervals (3∗1000 mg), methylprednisolone 1 mg/kg/day, and topical steroid drops once every 2 h.

**Figure 3:**
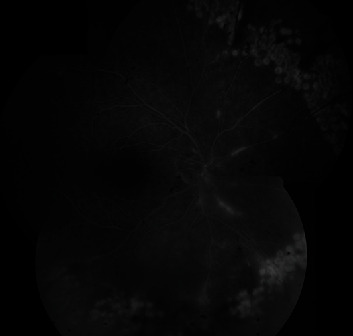
Fundus fluorescein angiography montage image (Canon 60UVi, Tokyo, Japan) showing ischemic areas in the laser periphery, leakage in the head of the optic disk, and vasculitis.

Visual acuity remained at the same level at the control examination one week later. Three hundred sixty-degree laser photocoagulation was applied to the posterior margin of the necrotized area in the peripheral fundus. The methylprednisolone dosage began being tapered to 8 mg once every three days, while valacyclovir and topical steroid therapy were maintained at the same dosages. Fluorescein angiography (Canon 60UVi, Tokyo, Japan) performed five days after laser photocoagulation revealed that laser application to the rear of the peripheral ischemic area had been effective, and leakage was detected in the optic disk (Figure 3).

**Figure 4:**
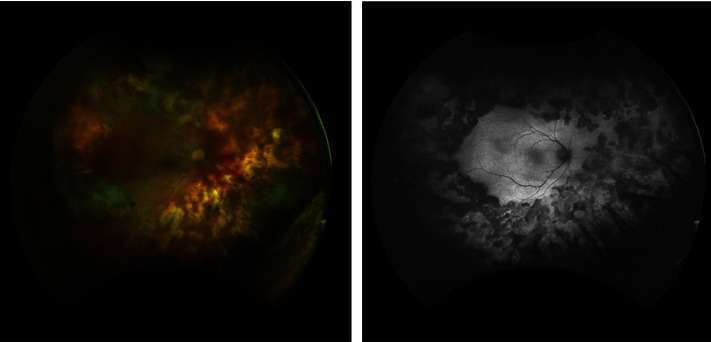
Wide-angle fundus photograph and fundus autofluorescence (FAF) images (Optos PLC, Dunfermline, UK). The laser photocoagulation therapy was effective, and no pathology was observed in the retina.

Visual acuity using a Snellen chart at control examination after one month was 0.5, and the anterior segment reaction and fundus examination findings had regressed; valacyclovir was reduced to 2∗1000 mg due to diffuse eruption on the patient's body. Methylprednisolone continued to be tapered, and topical steroid therapy was reduced. At control examination on the sixth week, the patient's visual acuity using a Snellen chart was 10/10. The anterior segment findings had resolved entirely, fundus examination findings had regressed, and the retinal hemorrhage in the laser periphery had decreased. In addition, ghost vessels, perivascular sheathing, and mild vitreous condensation were present. Cystoid macular edema (CME) was detected at OCT evaluation. Treatment was modified to valacyclovir 2∗1000, nepafenac drops 4∗1, and oral indomethacin 2∗25 mg. At the end of the second month, the patient's vision had diminished to the hand movement level, and the retina was completely detached. In the peripheral retina, except for the superotemporal quadrant, there was grade B proliferative vitreoretinopathy (PVR) with prominent wrinkling of the inner retinal surface and increased vessel tortuosity on the diffuse retinal surface. In the inferonasal peripheral retina, several atrophic holes with irregular borders without operculum in front of and behind the laser field, horseshoe tears behind the laser field at 2 and 3 o'clock, and a well-circumscribed atrophic hole with operculum behind the laser field at 7 o'clock were present. The patient was started on single-dose 300 mg/day acetylsalicylic acid (ASA) therapy, maintained for three months. Pars plana vitrectomy and silicone tamponade were applied. The silicone was removed three months after detachment surgery, and cataract surgery was performed one month after that operation. Valacyclovir therapy was stopped at the end of nine months. The patient's visual acuity reached 0.3 at the end of 11 months, and no pathology was observed in the retina (Figure 4). No pathology was also detected in the other eye.

**Figure 5:**
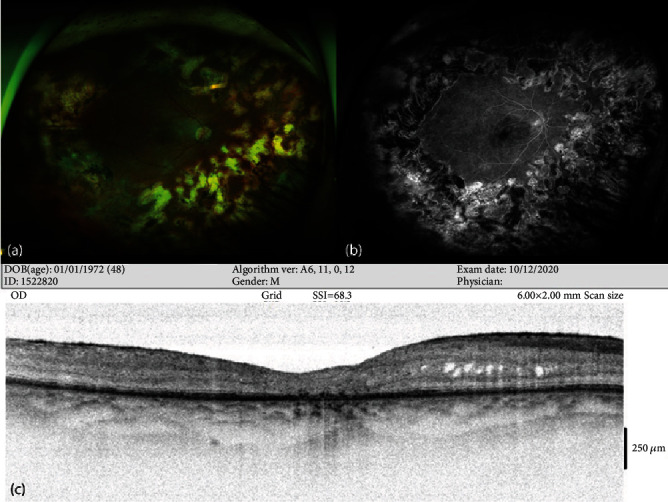
Right eye findings after 4.5 years: (a) wide-angle fundus photograph (Optos PLC, Dunfermline, UK), (b) fundus fluorescein angiography late phase image (Optos PLC, Dunfermline, UK), and (c) macular OCT image (Optovue RTVue, RT 100, software version 6.3, Optovue, CA, USA), atrophy in the outer retinal layers, irregularity in the ellipsoid zone, cystoid macular edema, and epiretinal membrane.

On the 13^th^ month of follow-up, the patient represented to our clinic due to mild redness and pain in the same eye. Visual acuity on a Snellen chart was 0.1, 1+ anterior chamber reaction and smooth-rounded keratic precipitates were present in the anterior segment, and posterior capsule opacities had developed. No activation was detected in retinal findings. The patient was started on 2∗500 mg valacyclovir, nepafenac drops 4∗1, and dexamethasone drops 4∗1. Right eye Nd-YAG laser capsulotomy was performed after one month. Valacyclovir was reduced to 1∗500 mg following laser therapy and was discontinued after one month. Visual acuity using a Snellen chart increased to 0.3 at the 16^th^ month of follow-up, and no findings of inflammation were observed on the anterior segment or fundus. The patient represented to our clinic due to mild blurred vision one month after that examination, at which +1 anterior chamber reaction in the anterior segment and mild condensation in the vitreous were observed. Valacyclovir was restarted at 2∗500 mg. In order to avoid the tendency to recurrence of anterior segment inflammation when valacyclovir was stopped, we recommended that treatment be maintained at this dose for at least six months. Visual acuity on a Snellen chart following approximately the two-year follow-up was 0.4, and no recurrence was observed. Valacyclovir therapy was maintained at 1∗500 mg and was stopped after three months. No kidney function disorder or additional side effect, other than cutaneous eruption during the initial high-dose therapy, was observed during valacyclovir therapy. The patient was followed up for a total of 4.5 years, and no recurrence or other ocular involvement was observed after 1.5 years (Figures 5(a) and 5(b)). Although an epiretinal membrane, minimal cystoid changes, atrophy in the outer retinal layers, and impaired integrity of the ellipsoid zone were determined on macular OCT at the final examination (Figure 5(c)), the best-corrected visual acuity on a Snellen chart remained stable at 0.3.

## 3. Discussion

Acute retinal necrosis is a rare, vision-threatening, and difficult to treat retinal pathology. Oral valacyclovir has recently been preferred to intravenous acyclovir in treatment, the aim being to reduce both drug-related side effects and hospitalization rates. Advantages such as the high bioavailability of oral valacyclovir, the fact that it can achieve similar plasma concentrations to those of intravenous acyclovir, and that it involves lower costs and outpatient treatment have resulted in increasing recent use in the treatment of acute retinal necrosis [11, 15, 16]. Several small case-number studies in recent years have shown that oral valacyclovir produces good visual and anatomical results [14, 17–19]. One retrospective study comparing intravenous acyclovir and oral valacyclovir reported similar visual acuity and retinal detachment rates in both groups, consistent with Tibbetts et al. [9, 12]. However, it is difficult to compare the studies due to their retrospective natures and their being in a case report or case series form, fundamental differences in characteristics, and their being performed at different times (in the 1980s and 1990s for intravenous therapy and the 2000s for oral therapy). Nonetheless, studies have concluded that intravenous acyclovir and oral valacyclovir therapies achieve similar outcomes in terms of reducing ocular involvement rates and achieving regression of retinal pathologies [11]. We employed oral valacyclovir therapy in the present case and achieved a stable outcome with ambulatory vision.

Several studies have shown that similar plasma concentrations are achieved with 1000 mg oral valacyclovir and 5 mg/kg or 350 mg intravenous acyclovir [15, 20]. Huynh et al. compared vitreous drug concentrations and plasma concentrations following oral valacyclovir therapy in 10 noninflamed eyes undergoing elective vitrectomy. Those authors reported that oral 1000 mg application every 8 h achieved a half maximal inhibitor concentration (IC₅₀) for VZV, HSV-1, and HSV-2 [21]. Liu et al. compared different dosages of oral valacyclovir and reported that oral valacyclovir 1500 mg three times daily resulted in similar outcomes to 700 mg intravenous acyclovir three times daily [22]. Although the therapeutic intravenous acyclovir dosage has been standardized, there are still differences in the literature regarding the application of oral valacyclovir at therapeutic doses. Some studies have elected to apply 1000 mg three times daily [18, 19], while others have applied 2000 mg three times daily [12, 14]. There is also no consensus in previous studies regarding the duration of treatment. Age, additional diseases, and compliance with treatment have been described as factors affecting the duration, and individualized treatment regimens have been applied accordingly. While the duration of treatment may be only two weeks for some patients, it may last for months in others [6, 8, 10]. We also started oral valacyclovir therapy at 1000 mg three times daily in the present case but revised this to 1000 mg twice daily due to eruption in the first month. Treatment was stopped after nine months, but when the patient twice presented with findings of mild anterior uveitis, we decided to continue with long-term treatment and only discontinued antiviral therapy after 1.5 years. We encountered no recurrence, inflammatory findings, or additional retinal pathologies at subsequent follow-ups over three years.

Although systemic antiviral therapy has been reported to reduce contralateral eye involvement, severe loss of vision and retinal detachment rates have been reported in the affected eye [3, 4, 7, 9]. Retinal detachment is one of the most frequent complications of acute retinal necrosis, generally appearing in the first three months after diagnosis. The prevalence of detachment ranges in previous studies between 41% and 85% [3, 4, 8, 9, 23–26]. Laser photocoagulation to the posterior of the necrosed area is recommended in order to prevent retinal detachment. However, while some studies have described laser as beneficial and reducing the development of retinal detachment [4, 27, 28], others have reported no benefit [9, 12, 24, 29].

Studies have also recommended systemic and/or local steroid therapy and antiplatelet agents to reduce the damaging effect of host immunity in addition to systemic antiviral therapy and early pars plana vitrectomy to prevent retinal detachment [3, 9] Recently published case series have also shown that intravitreal foscarnet applied in addition to systemic antiviral therapy reduces the risk of severe vision loss and development of retinal detachment [30–32].

The most important outcome in patients undergoing acute retinal necrosis is the inability to preserve useful visual acuity. Several studies have reported visual acuity levels of 20/200 or below in 50% of cases [4, 7, 24]. Loss of visual acuity has been linked to low initial visual acuity, development of retinal detachment, and late initiation of antiviral therapy [12].

In addition to retinal detachment in acute retinal necrosis, complications such as cataract, epiretinal membrane, cystoid macular edema, macular ischemia, recurrent anterior uveitis, chronic vitritis, optic neuropathy, optic atrophy, hypotonia, phthisis, rubeosis, and glaucoma may also develop [11, 33]. We observed consecutive retinal detachment, cataract, cystoid macular edema, and epiretinal membrane development in this case followed up for 4.5 years.

Due to its rarity, there is no standard therapeutic plan for acute retinal necrosis. However, the objective is to achieve early regression of the disease and to prevent involvement of the contralateral eye through early diagnosis and treatment. Achieving optimal visual acuity, the prime aim is possible with early diagnosis, appropriate treatment, and accurate complication management.

In conclusion, as reported in the literature and observed in the present case, since complications may occur at any time, frequent follow-up and an effective therapeutic approach may permit the preservation of useful vision in patients with acute retinal necrosis.
